# The transcriptome of *lae1* mutants of *Trichoderma reesei* cultivated at constant growth rates reveals new targets of LAE1 function

**DOI:** 10.1186/1471-2164-15-447

**Published:** 2014-06-09

**Authors:** Erzsébet Fekete, Levente Karaffa, Razieh Karimi Aghcheh, Zoltán Németh, Éva Fekete, Anita Orosz, Melinda Paholcsek, Anikó Stágel, Christian P Kubicek

**Affiliations:** Department of Biochemical Engineering, Faculty of Science and Technology, University of Debrecen, Egyetem tér 1, H-4032 Debrecen, Hungary; Institute of Chemical Engineering, University of Technology of Vienna, Gumpendorferstrasse 1a, A-1060 Vienna, Austria; Department of Human Genetics, Faculty of Medicine, University of Debrecen, Nagyerdei krt. 98, H-4032 Debrecen, Hungary; Roche Hungary Ltd, Edison u. 1, H-2040 Budaörs, Hungary; Austrian Center of Industrial Biotechnology (ACIB), Petersgasse 12, A-8010 Graz, Austria

**Keywords:** *Trichoderma reesei*, Microarray, Chemostat, LAE1, Growth rate, Transcriptome

## Abstract

**Background:**

The putative methyltransferase LaeA is a global regulator that affects the expression of multiple secondary metabolite gene clusters in several fungi. In *Trichoderma reesei*, its ortholog LAE1 appears to predominantly regulate genes involved in increasing competitive fitness in its environment, including expression of cellulases and polysaccharide hydrolases. A drawback in all studies related to LaeA/LAE1 function so far, however, is that the respective loss-of-function and overexpressing mutants display different growth rates. Thus some of the properties attributed to LaeA/LAE1 could be simply due to changes of the growth rate.

**Results:**

We cultivated *T. reesei*, a *Δlae1* mutant and a *lae1*-overexpressing strain in chemostats on glucose at two different growth rates (0.075 and 0.020 h^-1^) which resemble growth rates at repressing and derepressing conditions, respectively. Under these conditions, the effect of modulating LAE1 expression was mainly visible in the *Δlae1* mutant, whereas the overexpressing strain showed little differences to the parent strain. The effect on the expression of some gene categories identified earlier (polyketide synthases, heterokaryon incompatibility proteins, PTH11-receptors) was confirmed, but in addition GCN5-N-acetyltransferases, amino acid permeases and flavin monooxygenases were identified as so far unknown major targets of LAE1 action. LAE1 was also shown to interfere with the regulation of expression of several genes by the growth rate. About a tenth of the genes differentially expressed in the *Δlae1* mutant under either growth condition were found to be clustered in the genome, but no specific gene group was associated with this phenomenon.

**Conclusions:**

Our data show that – using *T. reesei* LAE1 as a model - the investigation of transcriptome in regulatory mutants at constant growth rates leads to new insights into the physiological roles of the respective regulator.

**Electronic supplementary material:**

The online version of this article (doi:10.1186/1471-2164-15-447) contains supplementary material, which is available to authorized users.

## Background

Reverse genetics relies on the use of molecular biological methods to discover the function of a gene by analyzing the phenotypic effects that result from a manipulation of its function. While this approach mostly leads to valid information about the role of a gene in the physiology of the respective organism, some caveats are to be applied when these mutants are studied by –omics techniques: many gene mutants also display altered growth rates, and thus studying their gene or protein expression on plates or in batch cultures will inevitably also identify genes whose expression is controlled only by the growth rate itself. This may lead to flawed interpretations as to the potential targets of the investigated genes, particularly in the case of regulatory genes.

The *Aspergillus nidulans* LaeA protein, a putative *S*-adenosylmethionine-dependent (SAM) methyltransferase, was originally described as a global regulator of secondary metabolism [1]. LaeA is known to be a nuclear protein, and is localized to the nucleus [1–3]. It has therefore been suggested that it regulates transcription by protein lysine or protein arginine methyltransferase functions [1], and its function has putatively been linked to changes in chromatin structure [4]. Recent studies in *A. nidulans*, however, failed to identify a protein that is methylated by LaeA, but considerable automethylation was observed [5]. In addition, the LAE1- (the *Trichoderma reesei* orthologue of *Aspergillus* LaeA) effected transcriptome of *Trichoderma reesei* showed no correlation with histone methylation at the affected loci [6].

LaeA was later shown to also control developmental events, such as conidiation in numerous fungi [7–13], and fruiting body formation in *A. flavus*[14, 15]. We have recently shown that in *T. reesei,* LAE1 also controls conidiation, the expression of polysaccharide hydrolytic enzymes and of genes involved in eco-physiological fitness, whereas the effect on secondary metabolite biosynthesis was not that strongly pronounced as in the *Aspergilli*[16, 6]. Thus, current knowledge suggests that LaeA has a dynamic role in both fungal morphological and chemical development, and nutrition.

In *T. reesei*, *lae1* mutants are also differing in their growth rates, being slower in the loss-of-function mutant and faster in mutants overexpressing *lae1*[16]. We thus wondered to which extent our recent study on the LAE1-effected transcriptome of *T. reesei*[6] would have been biased by the different growth rates of the *lae1* mutants. This could have been particularly due for genes that are known to be expressed only at specific growth rates, such as e.g. those involved in secondary metabolite biosynthesis. Therefore, we chose to use chemostat cultures on D-glucose as a carbon source at two different growth rates (one carbon catabolite repressing and one carbon catabolite derepressing) to investigate the genome-wide changes in gene expression in relation to LAE1 function, using *Δlae1* and *OElae1* recombinant mutant strain of *T. reesei*.

## Results

### Verification of the experimental strategy

Constant-mass, carbon-limited, chemostat-type continuous fermentations were used to cultivate the parent strain *T. reesei* QM 9414, and the *Δlae1* and *OElae1* mutant strains derived from it on glucose (1%, w/v) as a sole carbon source at two different dilution rates (D = 0.075 h^-1^ and D = 0.020 h^-1^). These two dilution rates (henceforth referred to as “high” and “low” growth rate) have previously been shown to represent a state of carbon catabolite repression and of carbon catabolite derepression, respectively, in *T. reesei* and also in *A. nidulans*[17–19]. Cultures were grown batchwise for 24 hours after inoculation. At the first 6–7 residence times of the continuous cultivations, gradually attenuating oscillation of the specific biomass production occurred [20] after which the oscillation decreased to a non-significant level. The steady-state biomass concentration was 1.49 ± 0.11 g/L in all cultures irrespective of the dilution rate. By feeding the cultures with a medium containing 3 g/L D-glucose as a sole carbon source (see Methods section), this biomass data resulted in a calculated growth yield (grams of biomass formed per gram of carbon source consumed) of between 46 and 53% for all cultures, which correlates well with previously published data from various fungal D-glucose-limited cultures (see [20] for references), and with our previous studies on *T. reesei* steady-states [17, 18]. The residual glucose concentration was between 0.03 and 0.05 mM at both 0.075 and 0.020 h^-1^. These values correlate well with the affinities of the high affinity hexose transporters of filamentous fungi [21], and thus prove that our cultures were indeed glucose-limited. At 0.02 h^-1^ the low dilution rate, some conidiospore formation was visible in the parent strain but not at the *Δlae1*-mutant. In addition, both strains exhibited a small degree of pellet formation although the overwhelming majority of cells displayed filamentous morphology at 0.075 h^-1^. In summary, while there were some minor morphological differences between the two strains at the two dilution rates applied, they unlikely affected the general experimental strategy. We thus considered the system appropriate for the purpose of this study.

### LAE1 loss of function is more dominant at high growth rates

1069 of the 9123 genes that were used in the array showed an at least 2-fold up- or downregulation (with p <0.05) in *T. reesei Δlae1* when compared to the parent strain QM 9414 (Table 1; Additional file 1: Table S1). Of these, only 182 genes were >2-fold regulated both at 0.075 and 0.02 h^-1^, respectively. The highest number of genes was encountered when the *Δlae1* mutant was compared to the parent strain QM9414 at 0.075 h^-1^ (930 vs. 321 genes at high vs. low growth rate). Genes that were downregulated in the *Δlae1* strain accounted for the minor fraction in both cases (43% and 36% at high and low growth rates, respectively).

**Table 1:** **Number of genes affected by** ***lae1*** **manipulation in** ***T. reesei*** **at the two growth rates in comparison to the parent strain QM 9414**

Strain comparison	D [h^-1^]	Total	Upregulated			Downregulated		
			All genes*	Orphan genes	Unknown genes	All genes*	Orphan genes	Unknown genes
∆*lae1* vs. QM 9414	0.075	**1069**	**523**	**33**	**193**	**407**	**16**	**138**
∆*lae1* vs. QM 9414	0.020		**203**	**10**	**79**	**118**	**9**	**40**
OE*lae1* vs. QM 9414	0.075	**113**	**53**		**22**	**36**	**1**	**13**
OE*lae1* vs. QM 9414	0.020		**11**		**4**	**17**	**2**	**3**

*including unknown and orphan genes.

Only a considerably smaller number of genes (113) was differently regulated between the *OElae1* mutant and the parent strain (89 and 28 genes at high and low growth rate, respectively, with 4 genes being affected at both growth rates). Genes that were downregulated accounted for a minor fraction at the high growth rate, but accounted for 61% at the lower growth rate (Table 1; Additional file 2: Table S2). The validity of the array data was approved by qPCR analysis of the expression of a small number of genes (Additional file 3: Table S3).

**Figure 1:**
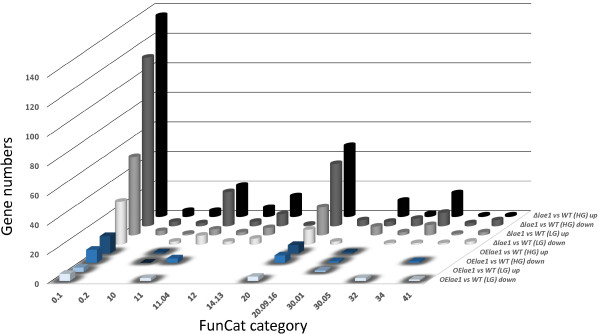
**Expression of genes belonging to selected FunCat groups in** ***T. reesei Δlae1*** **(** ***ΔLAE1*** **) and** ***T. reesei OElae1*** **(** ***OElae1*** **) during cultivation at 0.075 (HG) and 0.02 (LG) h** ^**-1**^ **, respectively.** “Up” means 2-fold upregulation, “down” 2-fold downregulation when compared to *T. reesei* QM9414 (*WT*). FunCat categories shown are: 01, metabolism; 02, energy generation; 10, cell cycle and DNA processing; 11, transcription; 11.4, RNA processing; 12, protein synthesis; 14.13, protein degradation; 20, transport; 20.09.16, cellular export and secretion; 30.01, intracellular signalling; 30.5, transmembrane signaling; 32, cell rescue and defense; 34, interaction with cellular environment; 41, development.

Under all conditions (except for *OElae1* at low growth rates, but this is likely due to the small gene number), more than a third of all genes encoded unknown proteins (i.e. such that shared orthologs in at least some other *Pezizomycota*) or were orphan genes (Table 1). In order to assess the global changes in gene expression brought about by the two *lae1* mutations, those genes for which a function was known or can be predicted were classified according to the FunCat categorization [22]; Figure 1. Of these, the majority of genes belonged to category “metabolism”, followed by “cellular transport” and “transcription”. However, none of the categories exhibited a statistically significant difference over another (p > 0.05 in all cases) when the number of genes in the individual categories was related to the total number of genes that were differently expressed under any of these conditions, This indicates that the cellular effect of the two *lae1* mutations is not reflected by changes in the expression of one general gene category.

Since the effect of LAE1 modulation may rather be reflected in the expression of genes encoding FunCat subcategories, we calculated the percentage of LAE1-effected genes of several subcategories in the complete (≥2-fold, p < 0.05) transcriptome and compared it to the percentage of these subcategories in the whole *T. reesei* genome. Thereby, some significant changes were observed (Table 2): genes encoding amino acid transporters, heteroincompatibility (HET) proteins, GCN5-N-acetyltransferases, and polyketide synthases (PKS) were significantly (p < 0.05) more abundant in the transcriptome affected in *Δlae1* at high growth rates than in the genome. A significantly higher number of genes belonging to the first three gene groups (*vide supra*) were differently expressed at the low growth rate. Genes encoding the PTH11-type receptors were significantly more abundant among genes that are higher expressed in the *Δlae1* mutant at high growth rates.

**Figure 2:**
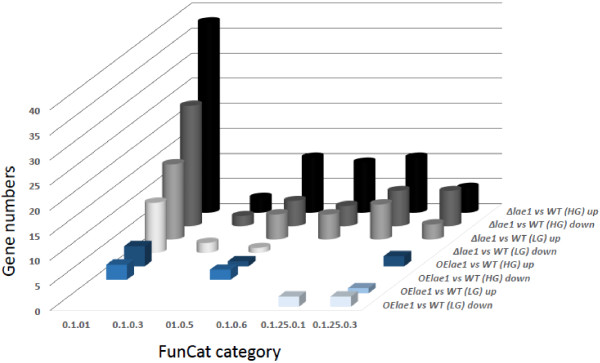
**Expression of genes belonging to metabolism-related FunCat groups in** ***T. reesei Δlae1*** **(** ***ΔLAE1*** **) and** ***T. reesei OElae1*** **(** ***OElae1*** **) during cultivation at 0.075 (HG) and 0.02 (LG) h** ^**-1**^ **, respectively.** “Up” means 2-fold upregulation, “down” 2-fold downregulation when compared to *T. reesei* QM9414 (*WT*). FunCat categories shown are: 01.01, amino acid metabolism; 01.03, nucleotide metabolism; 01.05, carbohydrate metabolism; 01.06, lipid metabolism; 01.25.01, extracellular carbohydrate degradation; 01.25.03, extracellular protein degradation.

In addition, LAE1-effected genes involved in metabolism were found to have a strong bias towards amino acid metabolism at the high growth rate, with the majority of them being downregulated (Figure 2). No significant changes in the expression of genes involved in intermediary metabolism (carbohydrate, lipid, amino acid and nucleotide metabolism) were noted at the low growth rate.

**Table 2:** **Comparison of some FunCat subcategory genes differentially expressed in the Δ** ***lae1*** **mutant compared to the** ***T. reesei*** **parent strain**

Function	FunCat	D = 0.075 [h^-1^]		D = 0.020 [h^-1^]		Present in the genome		p-value*
		Down	Up	Down	Up	Number	%	
Amino acid transporters	20_01_07	11	7	2	3	36	0.4	<0.01
C2H2 transcription factors	11_02_03_04**	3	6	0	1	54	0.6	
Cytochrome P450 proteins	32_07	2	6	1	0	61	0.7	
Flavin monooxygenases	16_21_05	2	5	0	4	43	0.5	
GCN5N-acetyltransferases	14_07	5	3	2	1	20	0.2	<0.02
Glycoside hydrolases	01_25_01	6	10	0	7	194	2.1	
Heteroincompatibility proteins	36_20	4	1	1	1	23	0.25	<0.05
Major facilitator superfamily	20_03	16	21	3	11	174	1.9	
Mitochondrial function	42_16	3	3	3	1	83	0.9	
NRPS	01_20_36	1	2	1	0	10	0.11	
PKS	01_20_05	2	1	0	0	12	0.13	<0.05
PTH11-receptors	30_05_02_24	2	3	0	1	24	0.25	
SSCRP	70_27	14	12	0	3	130	1.4	

*Only given for subcategories that were statistically supported to behave differently in the mutant.

**The subcategory specification refers to all transcription factors; C2H2 transcription factors do not have their own number.

With regards to secondary metabolism, only 3 of the 11 polyketide synthases (PKS), and 3 of the 10 non-ribosomal peptide synthases (NRPS) of *T. reesei* were shown to be affected by a loss of LAE1 function (Table 2), and with the exception of the NRPS Trire2:71005, which was downregulated in the *Δlae1* mutant at both growth rates, all the other genes were down- or upregulated only at D = 0.075 h^-1^. It is interesting to note that this number of affected PKS- and NRPS-encoding genes is nevertheless significantly higher than in our previous study [6], in which only a single NRPS gene (the siderophore synthase Trire2:69946) was found to be >2-fold downregulated in the Δlae1 mutant.

We also applied the same analysis to the genes which were at least 2-fold differently regulated between the *OElae1* mutant and the parent (Additional file 2: Table S2). However, no specific gene category or subcategory was found to accumulate to a significant extent.

### LAE1 loss-of-function affects growth rate regulation of gene expression

While the above data revealed genes that are affected by LAE1 function at a constant growth rate, we surmised that there may be also genes whose response to changes in the growth rate is dependent on LAE1. To this end we retrieved two data sets: one which contained genes that were growth rate dependent in *T. reesei* wild-type, but not in *Δlae1*, and another one which contained only genes whose expression was growth rate dependent in the *Δlae1* mutant strain, but not in the wild type. This revealed a total of 758 and 131 genes, respectively. Their annotation is given in Additional files 4 and 5: Tables S4 and S5: as can be seen, no specific gene category was significantly enriched in these two gene pools, indicating that the interference of LAE1 with growth-rate regulation affects specific genes rather than gene groups of similar function. A similar analysis was also made for the effect of *lae1* overexpression. Again, the number of genes was considerably smaller, and the majority of them consisted of unknown proteins (data not shown).

In addition, we also monitored those genes, whose growth-rate dependent expression in the *lae1* mutants was reverted, i.e. opposite as to the parent strain. 26 genes that were higher expressed at the high growth rate in *T. reesei* QM 9414 were found to be downregulated in *T. reesei Δlae1*, and 12 genes displayed the opposite trend. No specific FunCat category or subcategory was detected to be enriched in these two groups, which accounted for only a minor portion of the LAE1 effected transcriptome (Additional file 6: Table S6).

### The majority of genes effected by LAE1 are not clustered in the genome

We were further interested whether the genes affected in their expression by LAE1 function would show a clustering in the genome. To test this, we made use of REEF, a program that performs a serial hypergeometric distribution tests in a sliding window along the chromosomes implemented in Phyton program [23]. This program has the advantage that it uses statistical models to assess the significance of physical clusters detected. It has also previously shown to lead results consistent with other methods [24]. REEF detected 124 and 31 of the 930 and 321 genes differentially expressed between the *Δlae1* and the parent strain during cultivation at high and low growth rate, respectively, to be clustered in the genome (Table 3). The clustering was evident from a 2.2 and 2.6-fold enrichment in gene density in the transcripts from high and low growth rates, respectively, over the average distribution in the genome. 16 and 9 clusters were found for the transcripts from high and low growth rate, of which 4 were present under both conditions. 7 and 5, respectively, occurred with a 200,000 bp distance from one of the ends of the respective scaffold. This demonstrates that about a tenth of the genes affected in their expression by LAE1 loss-of-function are clustered in the genome.

**Table 3:** **Clustering of transcripts that are differentially expressed in Δ** ***lae1*** **vs the parent** ***T. reesei*** **QM 9414 at the two growth rates**

Scaffold	Size[Mbp]	D = 0.075 [h^-1^]				D = 0.020 [h^-1^]			
		Area [Mbp]		Genes clustered	Cluster size [genes]	Area [Mbp]		Genes clustered	Cluster size [genes]
		Begin	End			Begin	End		
1	2.76	2.26	2.43	9	50	2.45	2.57	3	39
2	2	0.04	0.15	7	28	0.04	0.21	4	44
		1.33	1.45	7	30				
3	1.91	0	0.16	9	54				
4	1.83	1.08	1.19	7	29	0.64	0.81	4	45
		1.15	1.26	7	30				
5	1.73	0.7	0.86	8	39	0.84	1.04	5	61
6	1.45	0.41	0.54	8	28	0.84	1.01	3	36
7	1.43	1.07	1.21	9	44	1.13	1.25	3	37
		1.21	1.33	8	31				
8	1.41	0.35	0.52	9	52				
		0.57	0.69	7	27				
		0.83	0.94	8	35				
10	1.16	0.92	1.04	7	28				
19	0.66	0.55	0.66	7	27	0.5	0.61	3	23
20	0.63					0.43	0.55	3	19
24	0.5	0.21	0.32	7	22				
25	0.44					0.16	0.3	3	35
Total:				124	554			31	339

We also evaluated the genes contained in these clusters, but no obvious trend was seen: gene groups more frequently occurring were identical to those more abundant in the genome. We also specifically looked for clustering of genes encoding secondary metabolites (NRPS, PKS) and potentially being involved in their biosynthesis (transcription factors, ABC transporters, cytochrome P450 proteins, aldo/keto reductases/dehydrogenases, flavoproteins) but obtained a similar result (data not shown).

## Discussion

To investigate the physiology of microbial mutant strains, they are usually cultivated on solid medium or in batch cultures. Under both conditions, individual nutrients will gradually become growth limiting, and cause changes in the actual specific growth rates, that in turn may profoundly influence the metabolism and consequently the physiology of the cell. While this fact has been well considered in studies of yeasts and bacteria, much fewer reports are available for the applications of methods that take the specific growth rate into consideration for filamentous fungal research [17–19, 25–28].

Because of this similarity to methyltransferases and its localization to the nucleus, LaeA/LAE1 has been postulated to regulate transcription by lysine or arginine protein methyltransferase functions [1, 29]. However, subsequent work showed that LAE1-modulated expression of genes in *T. reesei* did not correlate with corresponding changes in the histone methylation state at these gene loci [6]. In addition, Patananan et al. [5], using several different experimental approaches failed to identify a protein or other substrate in *A. nidulans* that becomes methylated by LaeA. Instead, LaeA was shown to methylate itself at M207, yet this methylation was not essential for LaeA function, and the corresponding M207 does also not occur in the *T. reesei* LAE1. All this evidence suggests that LaeA/LAE1 may exert its function by binding to other proteins rather than by methylating histones or other DNA-binding proteins.

LaeA has been isolated because of its role as a regulator of secondary metabolism in *A. nidulans*[1], and it was therefore surprising that we recently found only a single NRPS to be downregulated in the *Δlae1* mutant [6]. In the present study, two PKS and one NRPS genes were affected by lae1 loss-of function, and this effect occurred only at the high growth rate (or was much stronger in the case of the NRPS). The fact that our previous study was performed with lactose grown cells [6] may explain this discrepancy, because growth on lactose is characterized by a very slow growth rate. Thus the effect of LAE1 on secondary metabolism in *T. reesei* appears to be growth rate dependent, and more pronounced at the high growth rate. Veiga et al. [30] have also found a growth rate-dependency of LaeA and VeA function in *Penicillium chrysogenum*, although in the opposite direction.

Our data also showed that only a comparatively small percentage of the genes that are affected by LAE1 modulation (i.e. 10%) are in fact clustered in the genome. This value is much smaller than clustering percentages obtained for gene expression in *T. reesei* under other conditions such as conidiation [24]. While a similar clustering analysis of differently expressed genes in *T. reesei* growing on glycerol and glucose yielded still a lower value (4.3%; C.P.K., unpublished data), the significance of the 10% found in this study is unclear. In any case, the data show that most of the genes that are affected by LAE1 loss of function do not appear to be clustered in the genome of *T. reesei*.

However, the present study revealed a new level at which LaeA/LAE1 may control gene expression: we show that the expression of 50% of the GCN5-N-acetyltransferases (GNATs) present in the *T. reesei* genome is altered in the *Δlae1* strain, most of them being downregulated. This effect of LAE1 has not been revealed in previous studies [6], probably due to the use of batch cultures or of different carbon sources. GNATs catalyze the transfer of the acetyl group from acetyl coenzyme A to a primary amine. While – despite of the similarity to the GCN5 protein of the SAGA complex [31] - some of them may thus fulfill metabolic functions, a subgroup of them is known to acetylate histones at specific lysine residues, a process that leads to transcriptional activation and has been implicated in chromatin assembly [32, 33]. Unfortunately, none of the *T. reesei* GNATs has so far been functionally investigated, but one – Trire2:120120 – has been shown to be induced on the cellulase inducing carbon sources cellulose [34, 35] and lactose [36], is downregulated in *T. reesei Δlae1* strain [6] and its overexpression in *T. reesei* leads to a twofold stimulation of cellulase production. This gene is, however, not expressed on glucose and was therefore not detected under the conditions of this work. However, the above noted correlation between LAE1 function and action of the GNAT Trire2:120120 may also be valid for several of the GNATs detected to be LAE1-dependent in this work. In *A. nidulans*, LaeA function has recently also been linked to histone acetylation: in a multicopy suppressor screen for genes capable of restoring secondary metabolite production in an *A. nidulans ΔlaeA* mutant, the histone acetyltransferase EsaA was identified [37]. However, EsaA binds to the target histone by a chromo-domain and is a member of the Myb_Cef protein family (Pfam 11831), and not a GNAT. Thus, a direct link between GNATs and LaeA/LAE1, which may explain the postulated LaeA-dependent chromatin modification [29], still requires scientific testing. Interestingly, an orthologue of *S. cerevisiae* SPT10 – an acetyltransferase that directly activates the transcription of histone genes, and whose function is essential for normal cell division [38] – is also strongly downregulated in the *T. reesei Δlae1* mutant. Its downregulation in *T. reesei Δlae1* may be a factor contributing to the slower growth observed in this mutant. We should also like to note that the other observed effects such as changes in the growth-rate dependent regulation in the *Δlae1* strain would be fully compatible with an action of LAE1 via GNATs, because acetylation by them can also lead to repression of gene expression [39].

Another group of genes that were significantly influenced by LAE1 in this study – but not detected to be affected previously [6] – were amino acid transporters. These transporters can reliably be predicted by the presence of a common structural motif consisting of 12 alpha-helical putative transmembrane segments and cytoplasmically located N- and C-terminal hydrophilic regions, and belong to the amino acid/polyamine organocation superfamily [40]. In yeasts, they are usually absent during growth on an inorganic nitrogen source but upregulated once organic nitrogen becomes available, using transcriptional [41] and/or posttranscriptional mechanisms [42]. In contrast, the present study shows that these amino acid transporters are expressed in *T. reesei* during growth on an inorganic nitrogen source. These finding is also consistent with the observation that *T. reesei* prefers amino acids as carbon sources when grown in the presence of cellulose or lactose and amino acid mixtures [43], and thus regulation of expression of these permease genes still deserves attention. Interestingly, there is now emerging evidence that amino acid uptake in yeast is regulated by GNAT-dependent histone acetylation [44, 45], which would fit to the above supposed role of LAE1 in histone acetylation. We also noted that the effect of LAE1 on amino acid permeases is paralleled by a severe downregulation of a significant number of genes involved in amino acid metabolism in *T. reesei Δlae1* (mainly at the high growth rate), and the above findings of regulation of amino acid uptake by LAE1 can therefore be extended to a general effect on amino acid metabolism. Also, some extracellular proteases and oligopeptide transporters were effected by LAE1 (cf. Additional file 1: Table S1), but these two groups as a whole remained not significantly affected.

Most of the other FunCat groups found to be influenced by LAE1 function were already identified in our earlier study, which used lactose as a carbon source to induce cellulase gene expression [6]. Yet differences in the numbers of genes in the individual groups were noted. It is unclear, however, whether these observations are growth rate- or carbon source-specific.

## Conclusions

In this study, we used growth at controlled growth rates on glucose as a limiting carbon source to study the changes in gene expression effected by the protein methyltransferase LAE1 in *T. reesei* at a genome wide level. The corresponding results confirm several previously detected targets, but also reveal new findings: on one hand, the effect of LAE1 becomes mainly visible upon its deletion whereas overexpression has only a little effect. Also genomic clustering of the *lae1*-effected transcripts was observed to a much lower extent than previously [6], and is thus either due to the use of glucose as a carbon source or the use of constant growth rates for comparison. However, we detect for the first time that *lae1* gene deletion affect the regulation of gene expression by the growth rate. The latter finding likely points to an indirect influence, and suggests that LAE1 influences the formation of both positive as well negative signals or regulators of the respective genes. On the other hand, additional LAE1 targets were obtained of which the GCN5-N-acetyltransferases may offer a new understanding of the mechanism of LAE1 action. Our data show that – using *T. reesei* LAE1 as a model – the investigation of the transcriptome in a regulatory mutant at constant growth rates may reveal new insights into the function of the respective gene.

## Methods

### Strains and cultivations

*T. reesei* QM9414 (ATCC 26921), a moderately cellulase-producing mutant, and the mutants *Δlae1* and *OElae1* prepared from it [6] was used throughout this work and kept on potato dextrose agar (Sigma, St. Louis, MO).

Chemostat-type continuous cultivations were performed and analyzed as described earlier [17]. Inoculum cultures were pre-grown in 500 ml Erlenmeyer flasks on a rotary shaker (250 r.p.m.) at 30°C in a medium described by Karaffa et al. [17] with D-glucose (10 g/L) and peptone (0.1 g/L) as carbon sources. The feeding medium [17] contained D-glucose at an initial concentration of 3 g/L, a value low enough to make the culture carbon-limited. Two to four separate steady-states (i.e., independently initiated and run continuous cultures at the constant-mass stage) were sampled and analysed for each dilution rate and fungal strain. Fungal biomass taken from the steady-state chemostat cultures (i. e., from ones that exhibited no changes in biomass dry weight in three successive samples taken over a period of three residence times, and had very little (0.03 – 0.05 mM) residual D-glucose left in the medium during the same period) were subjected to total RNA extraction (see below). RNA was subsequently divided for the procedures of the microarray and Real-Time PCR analysis, respectively. This way, mycelia growing under identical environmental (and consequently physiological) conditions were used for the two expression analysis methods.

### Analytical methods

Determination of the mycelial dry weight (DCW) and the assessment of residual glucose concentration in growth media occured as described earlier [46].

### Transcriptome analysis

Mycelia were harvested from steady-state glucose-limited cultures. Total RNAs from cultures with high and low dilution (=specific growth) rate were extracted using SV Total RNA Isolation System (Promega), according to the manufacturer’s instructions. cDNA synthesis, labelling and hybridization was performed by Roche NimbleGen (Roche-NimbleGen, Inc., Madison, WI, USA) with a high density oligonucleotide microarray using 60-mer probes representing the 9.129 genes of *T. reesei* as described by Metz et al. [24]. Microarray scanning, data acquisition and identification of probe sets showing a significant difference (p = 0.05) in expression level between the different conditions were performed essentially as described previously [24]. Genes were identified by the aid of a completely manually annotated *T. reesei* genome database proprietary to C.P.K. The Euclidean distance metric method, as implemented in DNASTAR v5.1.2. build 3 (DNAstar Inc., Madison, WI), was used for Hierarchical Clustering. Genes were then classified according to their major annotation in the MIPS Functional Catalogue [33]. To determine whether there were differences in the functional categories in each cluster, the distribution within each cluster was compared to the total distribution of all the annotated genes using independent chi-square tests. The microarray data and the related protocols are available at the GEO web site (http://www.ncbi.nlm.nih.gov/geo/) under accession number GSE55652.

### qPCR

DNase treated (DNase I, RNase free; Thermo Scientific) total RNA (1 μg) was reversely transcribed with the Transcriptor High Fidelity cDNA Synthesis kit (Roche), according to the manufacturer’s protocol with a combination (1:1) of the provided oligo-dT and random hexamer primers. Reactions were performed with ready-to-use LightCycler® 480 Probes Master mix (Roche), according to the manufacturer’s protocol. Primers, amplification efficiency and R-square values are given in Additional file 7: Table S7. Determination of the PCR efficiency was performed using triplicate reactions from a dilution series of cDNA, and the amplification efficiency then calculated from the given slopes in the Realplex v2.2 software. Expression ratios were calculated using REST© Software [30]. All samples were analyzed in two independent experiments with three replicates in each run.

### Analysis of genomic clustering of transcripts

*T. reesei* genes have not yet been mapped to chromosomes, but their appearance on genomic scaffolds is known. In order to identify whether the transcripts would be clustered to particular areas on these scaffolds, we aligned them onto an ordered list of genes on the individual scaffolds. We tested the presence of clustering of expressed genes by two methods: one employed REEF, a software that identifies genomic regions enriched in specific features, using test statistic based on the hypergeometric distribution applied genome-wide by using a sliding window approach and adopting the false discovery rate for controlling multiplicity [23]. The software, source code and documentation were downloaded from http://telethon.bio.unipd.it/bioinfo/reef/ . To use it for *T. reesei*, the scaffolds were treated as chromosomes. A window width of 100 kb, a shift of 10 kb and a threshold q-value of 0.05 were used. A minimum number of 7 and 3 clustered genes were used as a threshold for the analysis of expression at the high and low growth rate, respectively.

## Electronic supplementary material

Additional file 1: Table S1: Genes differentially regulated in *T. reesei* Δlae1 in comparison to the parent strain *T. reesei* QM 9414. (XLSX 73 KB)

Additional file 2: Table S2: Genes differentially regulated in *T. reesei* OElae1 in comparison to the parent strain *T. reesei* QM 9414. (XLSX 245 KB)

Additional file 3: Table S3: Quantitative expression patterns determined by qRT-PCR of selected genes. (DOC 54 KB)

Additional file 4: Table S4: Genes regulated by the growth rate in *T. reesei* Δlae1 in comparison to the parent strain *T. reesei* QM 9414. (XLSX 15 KB)

Additional file 5: Table S5: Genes regulated by the growth rate in the parent strain *T. reesei* QM 9414 in comparison to *T. reesei* Δlae1. (XLSX 39 KB)

Additional file 6: Table S6: Genes regulated in the opposite way in *T. reesei* Δlae1 and the parent strain *T. reesei* QM 9414. (XLSX 11 KB)

Additional file 7: Table S7: Primers, amplification efficiency and R-square values for the qPCR analysis. (DOC 42 KB)
