# Supplementary material for: The transcriptome of lae1 mutants of Trichoderma reesei cultivated at constant growth rates reveals new targets of LAE1 function
Source: BMC Genomics. 2014 Jun 9;15(1):447. doi: 10.1186/1471-2164-15-447 (PMC4061448; doi:10.1186/1471-2164-15-447)
Supplement: Supplementary file 3 — Additional file 3: Table S3: Quantitative expression patterns determined by qRT-PCR of selected genes. (DOC 54 KB) [file 12864_2014_6119_MOESM3_ESM.doc]

Supplementary Table 3: Quantitative expression patterns determined by qRT-PCR of selected genes. QM: wild-type strain; Δlae1: lae1 deletion mutant; H: high growth rate (0.075 h^-1^); L: low growth rate (0.020 h^-1^)

| **Protein ID** | **Encoded protein** | **D (h^-1^)** | **Strain comparison** | **Expression*** | **p value*** | **Arrays** | **qPCR** |
| --- | --- | --- | --- | --- | --- | --- | --- |
| 73250 | Coenzyme F420-dependent N5,N10-methylene tetrahydromethanopterin reductase | 0.075 | ∆lae1 vs. QM | 32.85 | 0.001 | DOWN | DOWN |
| 106161 | NADP-glutamate dehydrogenase | 0.075 | ∆lae1 vs. QM | 1.02 | 0.001 | DOWN | DOWN |
| 122271 | DAHP synthase ARO4 | 0.075 | ∆lae1 vs. QM | 11.8 | 0.001 | DOWN | DOWN |
| 66092 | GcvT, Glycine cleavage system T protein | 0.075 | ∆lae1 vs. QM | 63.63 | 0.001 | DOWN | DOWN |
| 75230 | ILVD-Dehydratase family | 0.075 | ∆lae1 vs. QM | 4.06 | 0.001 | DOWN | DOWN |
| 107494 | Glutamate decarboxylase and related proteins | 0.075 | ∆lae1 vs. QM | 3.6 | 0.001 | DOWN | DOWN |
| 41325 | Bifunctional carbamoylphosphate synthase/aspartate carbamoyltransferase | 0.075 | ∆lae1 vs. QM | 14.87 | 0.001 | DOWN | DOWN |
| 53267 | Nop10p family Nucleolar RNA-binding protein | 0.075 | ∆lae1 vs. QM | 3.62 | 0.001 | DOWN | DOWN |
| 78611 | Ribosomal protein S29 (S14 family) by homology to the corresponding protein of *N. crassa* | 0.075 | ∆lae1 vs. QM | 9.33 | 0.001 | DOWN | DOWN |
| 78683 | UTP5, encoding a component of the SSU processome | 0.075 | ∆lae1 vs. QM | 2.5 | 0.001 | DOWN | DOWN |
| 80685 | Cys/Met metabolism PLP-dependent enzyme | 0.075 | ∆lae1 vs. QM | 3.53 | 0.001 | DOWN | DOWN |
| 61420 | HisG ATP phosphoribosyltransferase | 0.075 | ∆lae1 vs. QM | 2.5 | 0.001 | DOWN | DOWN |
| 23184 | IlvH Acetolactate synthase small (regulatory) subunit | 0.075 | ∆lae1 vs. QM | 0.18 | 0.001 | DOWN | DOWN |
| 2392 | Translation initiation inhibitor, yjgF family | 0.075 | ∆lae1 vs. QM | 3.96 | 0.001 | DOWN | DOWN |
| 66819 | 4-hydroxyphenylpyruvate dioxygenase | 0.075/0.020 | QMH vs. QML | 69.06 | 0.001 | DOWN | DOWN |
| 57185 | C2H2 transcriptional regulator (amdA) | 0.075/0.020 | QMH vs. QML | 1.78 | 0.001 | DOWN | DOWN |
| 105106 | Catalase | 0.075/0.020 | QMH vs. QML | 4.00 | 0.001 | DOWN | DOWN |
| 60144 | Amino acid transporters | 0.075/0.020 | QMH vs. QML | 2.51 | 0.001 | UP | UP |
| 106116 | Amino acid transporters | 0.075/0.020 | QMH vs. QML | 238 | 0.001 | UP | UP |
| 73250 | Coenzyme F420-dependent N5,N10-methylene tetrahydromethanopterin reductase | 0.075/0.020 | QMH vs. QML | 286 | 0.001 | UP | UP |

* Expression and p-values relate to the qPCR results.
