# Supplementary material for: The transcriptome of lae1 mutants of Trichoderma reesei cultivated at constant growth rates reveals new targets of LAE1 function
Source: BMC Genomics. 2014 Jun 9;15(1):447. doi: 10.1186/1471-2164-15-447 (PMC4061448; doi:10.1186/1471-2164-15-447)
Supplement: Supplementary file 7 — Additional file 7: Table S7: Primers, amplification efficiency and R-square values for the qPCR analysis. (DOC 42 KB) [file 12864_2014_6119_MOESM7_ESM.doc]

Supplementary Table S7: Primers, amplification efficiency and R-square values for the qPCR analysis

| **Protein ID** | **Oligonucleotide (5’-…- 3’)** | **UPL probe (Roche)** | **Amplification efficiency (%)** | **R^2^ Value** |
| --- | --- | --- | --- | --- |
| 23184 | 23184F: ccaaaagatccgcgagag  23184R: ttgtgggtgattgtgacgag | #89 | 1.79 | 0.98 |
| 41325 | 41325F: cgtctttctcacaagcgaca  41325R: tcgccgattgctacacct | #75 | 1.70 | 0.98 |
| 61420 | 61420F: agcctatccccgaaagtca  61420R: gaagagggcgtctgtcttgt | #25 | 1.75 | 0.97 |
| 66092 | 66092F: ggggagcaaagattgatgc  66092R: tgcccttctgcctgagac | #153 | 1.57 | 0.99 |
| 75230 | 75230F: atcgtggtcaactaccacagc  75230R: gccaaaggactcgaccag | #70 | 1.70 | 0.98 |
| 80685 | 80685F: gattccgacgatgaggagat  80685R: ccggcaccagtggtaact | #22 | 1.75 | 0.99 |
| 107494 | 107494F:gctcccttaaggagaagtgacc  107494R: gagtagacgccggtcgatatt | #70 | 1.70 | 0.96 |
| 122271 | 122271F: tgacattttcgacttgatgga  122271R: gccgcaaaatgtcctcatac | #153 | 1.72 | 0.99 |
| 78683 | 78683F: caacgacaccaactacggtct  78683R: atggccgtgttgagatcc | #70 | 1.56 | 0.97 |
| 2392 | 2392F: cctcgtgcccaacttctc  2392R: cggatctcgaggtactgctc | #25 | 1.72 | 0.98 |
| 53267 | 53267F: agcagcagcagaccatgag  53267R: gtcgaggctcttggacga | #153 | 1.84 | 0.97 |
| 78611 | 78611F: catcctcgagggtcgtga  78611R: tactttggccgctgcact | #153 | 1.67 | 0.98 |
| 57185 | 57185F: tgctttcttcttgacgctctc  57185R: cctcgaacaaggcaaacaat | #153 | 0.97 | 0.99 |
| 60144 | 60144F: tcaagatgcttctggtgctc  60144R: agggcccaacgtcgtaac | #25 | 0.95 | 0.99 |
| 66819 | 66819F: gcgataggattccgctactg  66819R: gccgtcttccagatactcgt | #81 | 0.92 | 0.99 |
| 73250 | 73250F: tcggaaatgggggaagat  73250R: atacccaatggcccaagaac | #44 | 0.995 | 0.97 |
| 105106 | 105106F: caagaccaggaggagagcat  105106R: ggagatgaggaccgatgtgt | #75 | 1.07 | 0.997 |
| 106116 | 106116F: atccaggtgggtgaagtcaa  106116R: gccgtagccgagcatatc | #44 | 0.85 | 0.99 |
| 106161 | 106161F: caacctgggagactgcaac  106161R: gacgttggtcatggcgtag | #81 | 1.01 | 0.9917 |
| tef1 | trire_tef1_F: tcaacggtgacaacatgctc  trire_tef1_R: ttctcccagcccttgtacc | #22 | 1 | 0.99 |
